# Efficient Serum-Free Rabies Virus Propagation Using BSR and Vero Cell Lines: A Comparative Evaluation of BioNOC II^®^ Macrocarriers in the BelloStage™-3000 Bioreactor Versus Conventional Microcarriers

**DOI:** 10.3390/biology14101455

**Published:** 2025-10-21

**Authors:** Zhanat Amanova, Zhanna Sametova, Sholpan Turyskeldy, Alina Kurmasheva, Ruslan Abitayev, Abdurakhman Ussembay, Zhanat Kondibayeva, Dariya Toktyrova, Dana Mazbayeva, Sergazy Nurabayev, Aslan Kerimbayev, Yerbol Bulatov

**Affiliations:** 1 Research Institute for Biological Safety Problems, National Holding “QazBioPharm”, Gvardeiskiy 080409, Kazakhstan; zh.sametova@biosafety.kz (Z.S.); sh.smankizi@biosafety.kz (S.T.); a.kurmasheva@biosafety.kz (A.K.); r.abitaev@biosafety.kz (R.A.); a.ussenbay@biosafety.kz (A.U.); zh.kondybaeva@biosafety.kz (Z.K.); d.toktirova@biosafety.kz (D.T.); d.mazbayeva@biosafety.kz (D.M.); s.nurabayev@biosafety.kz (S.N.); a.kerimbayev@biosafety.kz (A.K.); 2 Faculty of Biology and Biotechnology, Department of Biotechnology, Al-Farabi Kazakh National University, Almaty 050040, Kazakhstan

**Keywords:** rabies virus, bioreactor, BSR cell line, Vero cell line, serum-free culture medium, microcarrier, macrocarrier

## Abstract

**Simple Summary:**

Rabies is a deadly disease that can infect both animals and humans. One of the most reliable ways to prevent it is through the vaccination of animals. This study demonstrates an effective approach to culturing rabies virus using BSR and Vero cell lines. The cells were cultured in the serum-free nutrient medium OptiPRO™ SFM, which reduces the risk of microbial contamination. Two types of carriers were used for cell growth: BioNOC II^®^ macrocarriers and Cytodex 1 and Cytodex 3 microcarriers. Cultivation on macrocarriers was carried out in the BelloStage™-3000 bioreactor, which operates on the principle of “Tide motion”, ensuring gentle mixing and protecting the cells from damage. Cultivation on microcarriers was performed in flasks with a magnetic stirrer. The results showed that the use of macrocarriers in combination with the bioreactor system BelloStage™-3000 made it possible to obtain significantly more viral material in a shorter time. This approach makes vaccine production faster, safer, and more efficient. This is especially important for regions where rabies still poses a serious threat to the health of animals and humans.

**Abstract:**

The rabies virus remains a significant public health threat, particularly in regions with limited access to vaccination. This study shows that the BelloStage™-3000 bioreactor, operating on the “Tide Motion” principle, in combination with BioNOC^®^ II macrocarriers, ensures highly efficient rabies virus cultivation in BSR and Vero cells grown in serum-free OptiPRO™ SFM medium. This system supports effective cell attachment, formation of a dense and metabolically active cell layer, and reduces microbial contamination risks associated with serum-containing media. For comparison, rabies virus cultivation was also performed on Cytodex 1 and Cytodex 3 microcarriers in spinner flasks. The use of the BelloStage™-3000 bioreactor system with BelloCell™ 500A disposable vials and BioNOC II^®^ macrocarriers resulted in significantly higher virus titers compared to traditional Cytodex 1 and Cytodex 3 microcarrier culture systems. Thus, in the BSR cell culture, the maximum virus titer reached 5.6 × 10^8^ FFU/mL by day 4 of cultivation, which exceeded the titers obtained on Cytodex 1 and Cytodex 3 microcarriers by about 19.3-fold and 15.3-fold, respectively. A similar trend was observed for the Vero cell line: the peak titer was 2.0 × 10^8^ FFU/mL by day 5 of culturing, which was higher than the values obtained on Cytodex 1 and Cytodex 3 by about 14.0-fold and 9.6-fold, respectively. These findings demonstrate that the integrated use of BioNOC^®^ II macrocarriers, the BelloStage™-3000 bioreactor, and a serum-free medium provides a scalable, reproducible, and biosafe platform for rabies virus production, offering substantial advantages over traditional microcarrier-based systems.

## 1. Introduction

Rabies virus is caused by neurotropic viruses of the genus Lyssavirus of the family Rhabdoviridae in the order Mononegavirales and can be transmitted to all mammals [1,2]. The only reliable way to control rabies is specific prophylaxis, namely, vaccination of wild fauna, which represents the main reservoir of infection [3,4,5]. Despite ongoing prevention efforts, rabies remains endemic, particularly in developing countries, where both animal and human cases continue to occur [6,7,8].

The consequences of rabies extend beyond wildlife, as the disease is responsible for an estimated 59,000–69,000 human deaths annually, with the greatest burden in Asia and Africa, while also imposing substantial economic costs worldwide. Approximately 99% of human cases are acquired via the bite of an infected dog, rather than through exposure to the numerous wild animals that serve as viral reservoirs on different continents [9,10,11,12]. Kazakhstan provides a pertinent example of this dynamic, as rabies is reported annually in both domestic and wild animals, illustrating the ongoing regional epizootic situation and highlighting the importance of sustained surveillance and vaccination efforts [13].

The development of rabies vaccines, beginning with Pasteur’s pioneering work, laid the foundation for effective immunization. Although modern technologies provide new opportunities, mass vaccination of dogs and wild carnivores with currently available anti-rabies vaccines remains essential for reducing deaths in endemic regions. This integrated approach is crucial to achieving the WHO Global Strategic Plan’s goal of eliminating rabies-related deaths by 2030 [5,14].

In vaccine development, ensuring process stability and product quality is critical, with particular attention paid to culture media composition. Classical production often relies on animal-derived components such as serum, trypsin, and lactalbumin. While serum promotes cell growth, it has major drawbacks: undefined composition, risk of adventitious agents (e.g., endotoxins), variable cell performance, and high cost. Therefore, serum-free in vitro culture of animal cell lines is widely recognized as advantageous [15,16,17].

In Kazakhstan, most veterinary vaccines are produced using conventional serum-containing media and adherent growth platforms, such as roller bottles and cell factories. While effective, these methods are limited in scalability, expensive, and raise regulatory concerns due to the use of animal-derived components. Advanced tissue culture technologies, such as packed-bed bioreactors, offer a promising solution by enabling high-density cultures of adherent cells within a confined region of the reactor [18,19,20]. Although fixed-bed reactors have been employed in bioprocessing since the mid-20th century, the first single-use, fully integrated, high-cell-density systems were introduced only about a decade ago. Since then, research has increasingly focused on scaling traditional manufacturing processes to single-use fixed-bed vessels [21].

These approaches allow the elimination of animal-derived components, improve biosafety, and reduce production variability. Recent studies have demonstrated the feasibility of producing rabies virus in serum-free suspension systems or microcarrier-based platforms; however, data remain limited and are often not validated in fully integrated bioreactor systems [22,23,24,25,26,27,28,29]. In contrast to previous work, this study employs a fully serum-free medium and evaluates rabies virus production in BSR and Vero cell lines on BioNOC II^®^ macrocarriers within the BelloStage™-3000 bioreactor, thereby establishing a standardized and scalable process suitable for modern vaccine manufacturing.

In this study, we used the fixed rabies virus strain “Rabies virus fix/NIPBB/2024,” which had been adapted to BSR and Vero cell lines and further optimized for large-scale suspension culture aimed at animal rabies vaccine production. However, the scalability of the developed process for producing the animal rabies virus remains limited, as it relies on culture media containing animal-derived components and conventional cell culture methods such as cell factories and roller bottles. This factor reduces and complicates batch-to-batch reproducibility and may pose potential regulatory barriers in the future. To overcome these limitations, our objective was to develop an efficient and scalable cultivation platform based on BSR and Vero cell lines for the production of animal rabies virus. Furthermore, to streamline rabies virus cultivation, we employed a serum-free medium devoid of animal-derived components. Accordingly, this study aimed to enhance viral yield by employing an efficient substrate, optimizing conditions for viral replication, and simplifying the bioprocess using a serum-free medium and modern technological approaches.

Based on this objective, a comparative analysis of rabies virus replication in various culture media was conducted in suspensions of BSR and Vero cell lines using serum-free OptiPRO™ SFM medium (#12309019, Gibco™, Life Technologies, Grand Island, NY, USA), Cytodex 1 microcarriers (#GE17-0448-03, Cytiva, Uppsala, Sweden), Cytodex 3 (#GE17-0485-01, Cytiva, Uppsala, Sweden), and BioNOC II^®^ macrocarriers (Esco Micro Pte. Ltd., Singapore). The highest virus yield was observed in BelloCell^®^ 500A vials with BioNOC II^®^ macrocarriers. The process of culturing BSR and Vero cells and rabies virus on BioNOC II^®^ macrocarriers was tested in a BelloStage™-3000 tabletop bioreactor (Esco Micro Pte. Ltd., Singapore). Based on the results obtained, a protocol for the production of an animal rabies vaccine was developed.

## 2. Materials and Methods

### 2.1. Rabies Virus Strain, Cell Line, and Cell Culture Medium

The strain “Rabies virus fix/NIIPBB/2024”, a fixed strain specifically adapted to the BSR and Vero cell lines, was obtained from the Laboratory of Microorganism Collection of the Research Institute for Biological Safety Problems, Republic of Kazakhstan. The BSR and Vero cell lines, obtained from the Cell Biotechnology Laboratory of the Research Institute for Biological Safety Problems, Republic of Kazakhstan, was used as a substrate for rabies virus propagation and microtiter assay. Prior to use, the BSR and Vero cell lines were tested and confirmed to be free of mycoplasma. Cells were maintained in OptiPRO™ SFM medium (#12309019, Gibco, Life Technologies, Grand Island, NY, USA) supplemented with 100 U/mL penicillin, 100 μg/mL streptomycin, and L-glutamine (#25030081, Gibco, Life Technologies, Grand Island, NY, USA). All work with rabies virus was conducted under BSL-3 conditions with approval from the Research Institute for Biological Safety Problems, Republic of Kazakhstan.

### 2.2. Preparation of Microcarriers

Cytodex 1 (#GE17-0448-03, Cytiva, Uppsala, Sweden) and Cytodex 3 (#GE17-0485-01, Cytiva, Uppsala, Sweden) microcarriers were used in this study. The microcarriers were prepared according to the manufacturer’s instructions.

### 2.3. Cultivation of BSR and Vero Cells on a Techne Magnetic Stirrer

BSR and Vero cell lines were cultured on Cytodex 1 or Cytodex 3 microcarriers (2.0 g/L) in 250 mL Techne spinners (Bibby Scientific, Stone, UK) at 37 °C in a humidified incubator with 5% CO_2_. Spinners were inoculated at a density of ≥3.0 × 10^5^ cells/mL, and culture conditions (pH 7.0–7.4; glucose ≥ 1.0 g/L) were monitored daily. After 48 h, 60% of the medium was replaced with fresh medium. Cell growth dynamics were evaluated by daily sampling and determination of cell concentration. Cells were detached from microcarriers using 2 M NaCl, and viability was assessed by the trypan blue exclusion method with a TC20 automated cell counter (Bio-Rad Laboratories Pte. Ltd., Singapore).

Inoculum doses, stirring speeds, and glucose adjustments were based on preliminary optimization to ensure efficient virus replication, maintain cell viability, and support metabolic requirements during cultivation.

Note: Detailed operational parameters, including stirring speeds, are provided in Supplementary Materials.

### 2.4. The Process of Inoculation and Proliferation of the Rabies Virus on Microcarriers

When BSR and Vero cells reached 1.5–2.5 × 10^6^ cells/mL, two-thirds of the medium was replaced with fresh OptiPRO™ SFM containing the virus at 0.1 TCID_50_/mL. Virus adsorption was performed for 30 min, after which cultures were maintained at 37 °C with 5% CO_2_. After 48 h, the medium was refreshed. Cell concentration, pH (7.2–7.6), and glucose (>1.0 g/L) were monitored daily. Once 80–90% of cells were infected, the viral suspension was harvested by freeze–thaw.

Note: Detailed operational parameters, including stirring speeds, are provided in Supplementary Materials.

### 2.5. Cultivation of BSR and Vero Cells in the BelloStage™-3000 Bioreactor System

BSR and Vero cells were cultured in 300 cm^3^ flasks using OptiPRO™ SFM supplemented with L-glutamine. Cells were detached with 0.25% trypsin-EDTA, centrifuged, and resuspended at ≥1.5 × 10^7^ cells/mL. Suspensions (20 mL) were inoculated into BelloCell 500A flasks with BioNOC II^®^ macrocarriers pre-equilibrated in medium. Cells were allowed to attach at 37 °C for 5 h, after which 380 mL of fresh medium was added and flasks transferred to the BelloStage™-3000 system. Cultures were maintained with daily monitoring of cell density, pH (7.0–7.4), and glucose (>1.0 g/L), with medium replacement after 48 h.

Note: Detailed operational parameters are provided in Supplementary Materials.

### 2.6. Inoculation and Propagation of Rabies Virus on BioNOC II^®^ Macrocarriers

BSR and Vero cells were seeded at 1.5–3.0 × 10^8^ cells on BioNOC II^®^ macrocarriers, pre-washed with 500 mL of OptiPRO™ SFM. The cultures were inoculated with rabies virus (strain fix/NIIPBB/2024) at 0.1 TCID_50_/mL and incubated for 1 h at 37 °C in the BelloStage™-3000 system. pH (7.2–7.6) and glucose levels (≥1.0 g/L) were monitored daily.

Note: Detailed operational parameters are provided in Supplementary Materials.

### 2.7. Assessment of Cell Density on BioNOC II^®^ Macrocarriers

Cell density was determined using the method described in reference [30].

### 2.8. Glucose Level Monitoring

Glucose levels were monitored using the GlucCell™ system (Esco Micro Pte. Ltd., Singapore). When the glucose concentration in the growth medium of BSR or Vero cell lines dropped below 1.0 g/L, glucose (G8769-100ML, Sigma-Aldrich, St. Louis, MO, USA) was supplemented to restore the concentration to its initial range of 1.0–1.5 g/L. If the glucose concentration in the growth medium during virus replication dropped below 1.0 g/L, additional glucose was added to raise the level to 3.0 g/L, thereby ensuring optimal conditions for viral replication.

### 2.9. Monitoring of pH

The pH was measured using a pH meter following the method described in reference [30]. When a decrease in the culture medium pH was observed, it was adjusted by regulating the CO_2_ concentration in the incubator and periodically adding a 7.0% NaHCO_3_ solution (#144558, Sigma-Aldrich, St. Louis, MO, USA).

### 2.10. Monitoring of Cellular Infection

The progression of viral infection was assessed by direct immunofluorescence using FITC-conjugated monoclonal antibodies specific to the nucleocapsid protein of the rabies virus (Fujirebio Diagnostics, Inc., Malvern, PA, USA). Cells were fixed with 80% acetone, incubated with the antibodies, and examined by fluorescence microscopy. The extent of infection was evaluated by counting the number of fluorescent cells across multiple fields of view.

### 2.11. Monitoring of Virus Replication

Virus replication was assessed using a modified rapid fluorescent focus inhibition test (RFFIT) as described in reference [26], and the results were expressed in fluorescent focus units per milliliter (FFU/mL).

### 2.12. Statistical Analysis of Data

Statistical analysis was conducted using GraphPad Prism software version 8.0.1. Rabies virus titers obtained from different cultivation methods were compared using a two-tailed Student’s *t*-test. Differences were considered statistically significant at *p* < 0.05.

## 3. Results

### 3.1. Growth of BSR and Vero Cell Lines on Cytodex 1 and Cytodex 3 Microcarriers

**Figure 1:**
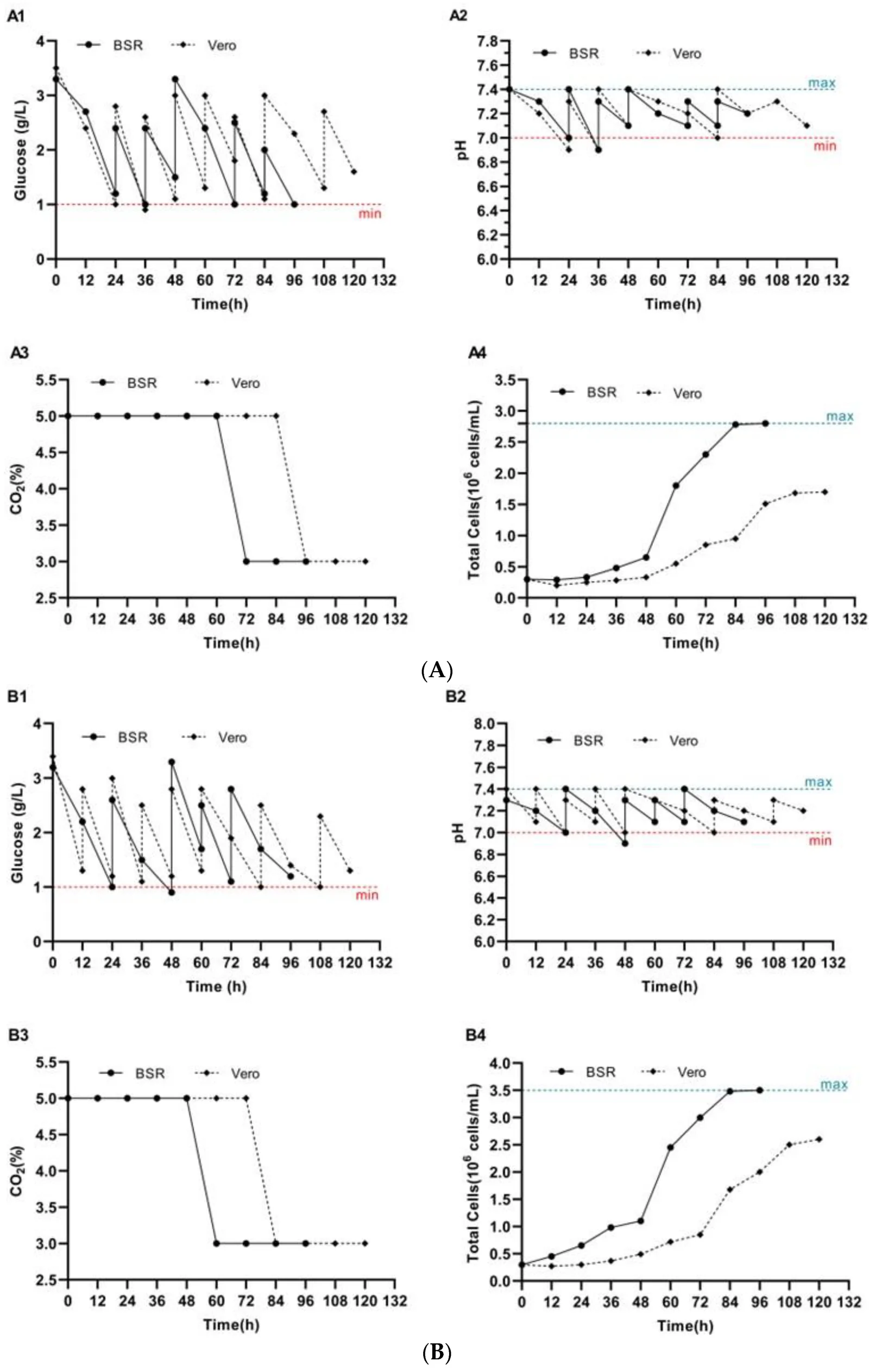
BSR and Vero cell growth on Cytodex 1 (**A**) and Cytodex 3 (**B**) microcarriers. Key cultivation parameters are presented: glucose concentration (**A1**,**B1**), pH of the nutrient medium (**A2**,**B2**), CO_2_ concentration in the incubator (**A3**,**B3**), and total cell density of BSR and Vero cells (**A4**,**B4**). In the graphs, the blue dotted line represents the upper limit (maximum), while the red dotted line indicates the lower limit (minimum).

The growth of BSR and Vero cells on Cytodex 1 and Cytodex 3 microcarriers was evaluated microscopically. Cell density was determined by detaching the cells from the microcarriers using a 2 M NaCl solution, followed by cell counting. Cell counting was performed using the TC20™ automated cell counter. The conducted studies revealed that BSR and Vero cells began attaching to the surface of Cytodex 1 and Cytodex 3 microcarriers as early as 18–24 h after seeding. By 96 h of cultivation, the surface coverage of Cytodex 1 by BSR cells reached 85–90%, and up to 95% for Cytodex 3. In comparison, Vero cells achieved up to 80% coverage on Cytodex 1 and up to 90% on Cytodex 3. The glucose level in the culture medium was maintained at an optimal concentration of no less than 1.0–1.5 g/L for both cell lines throughout the entire cultivation process (Figure 1(A1,B1)). The pH value was controlled and stabilized within the range of 7.0–7.4 (Figure 1(A2,B2)) by regulating the CO_2_ concentration in the incubator (Figure 1(A3,B3)) and through periodic supplementation with a 7.0% NaHCO_3_ solution.

The growth kinetics of BSR and Vero cells on Cytodex 1 and Cytodex 3 microcarriers were evaluated at 12-h intervals following the initial attachment phase (Figure 1(A4,B4)). The initial seeding density of BSR and Vero cells was 3.0 × 10^5^ cells/mL for both types of microcarriers. The maximum cell density for BSR cells was reached on day 4 of cultivation and amounted to 2.8 × 10^6^ cells/mL on Cytodex 1 and 3.5 × 10^6^ cells/mL on Cytodex 3. For Vero cells, the maximum density was observed on day 5 and reached 1.7 × 10^6^ cells/mL on Cytodex 1 and 2.6 × 10^6^ cells/mL on Cytodex 3, indicating higher proliferative activity on Cytodex 3 carriers (Figure 1(A4,B4)).

### 3.2. Reproduction of Rabies Virus on Microcarriers Cytodex 1 and Cytodex 3

**Figure 2:**
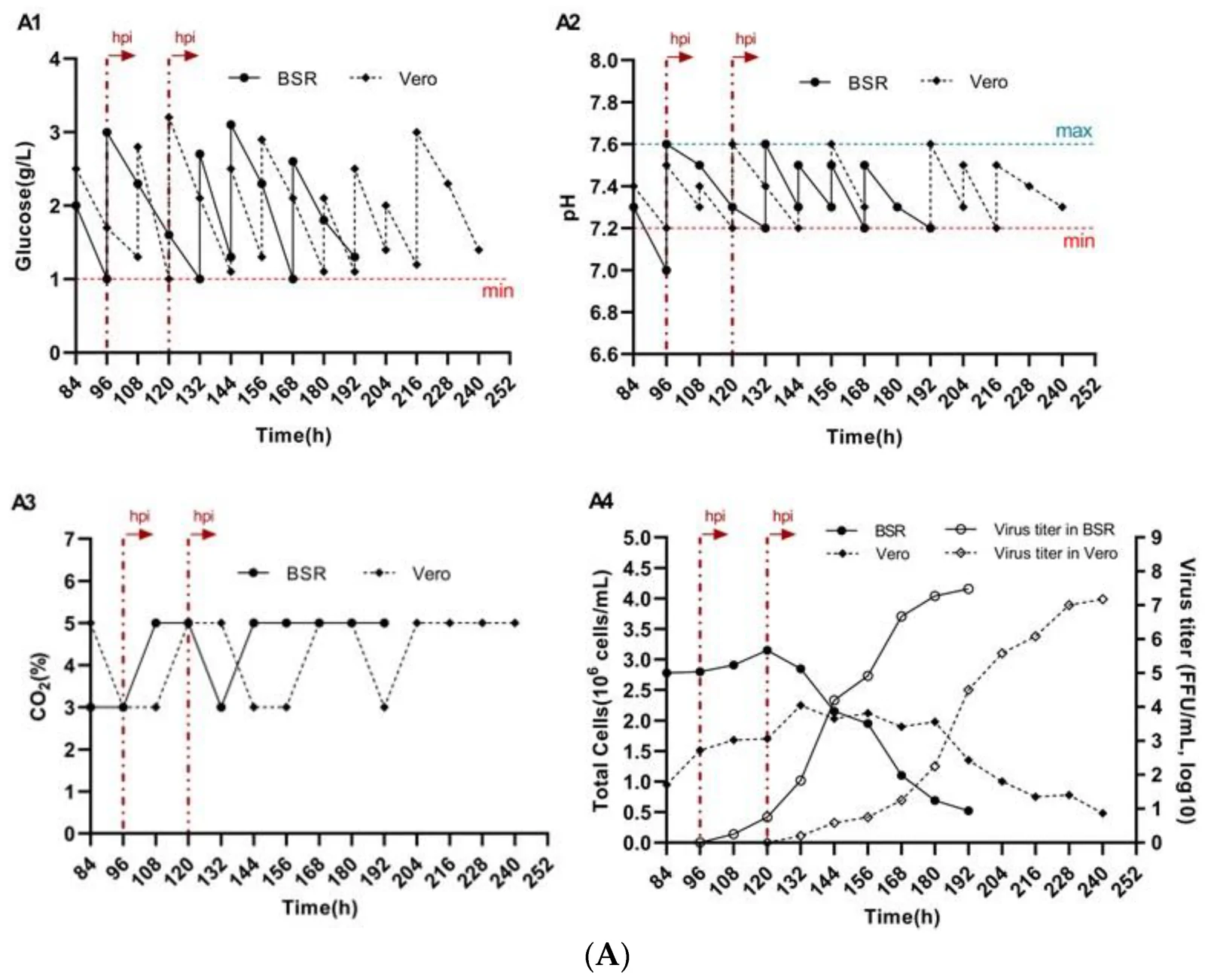

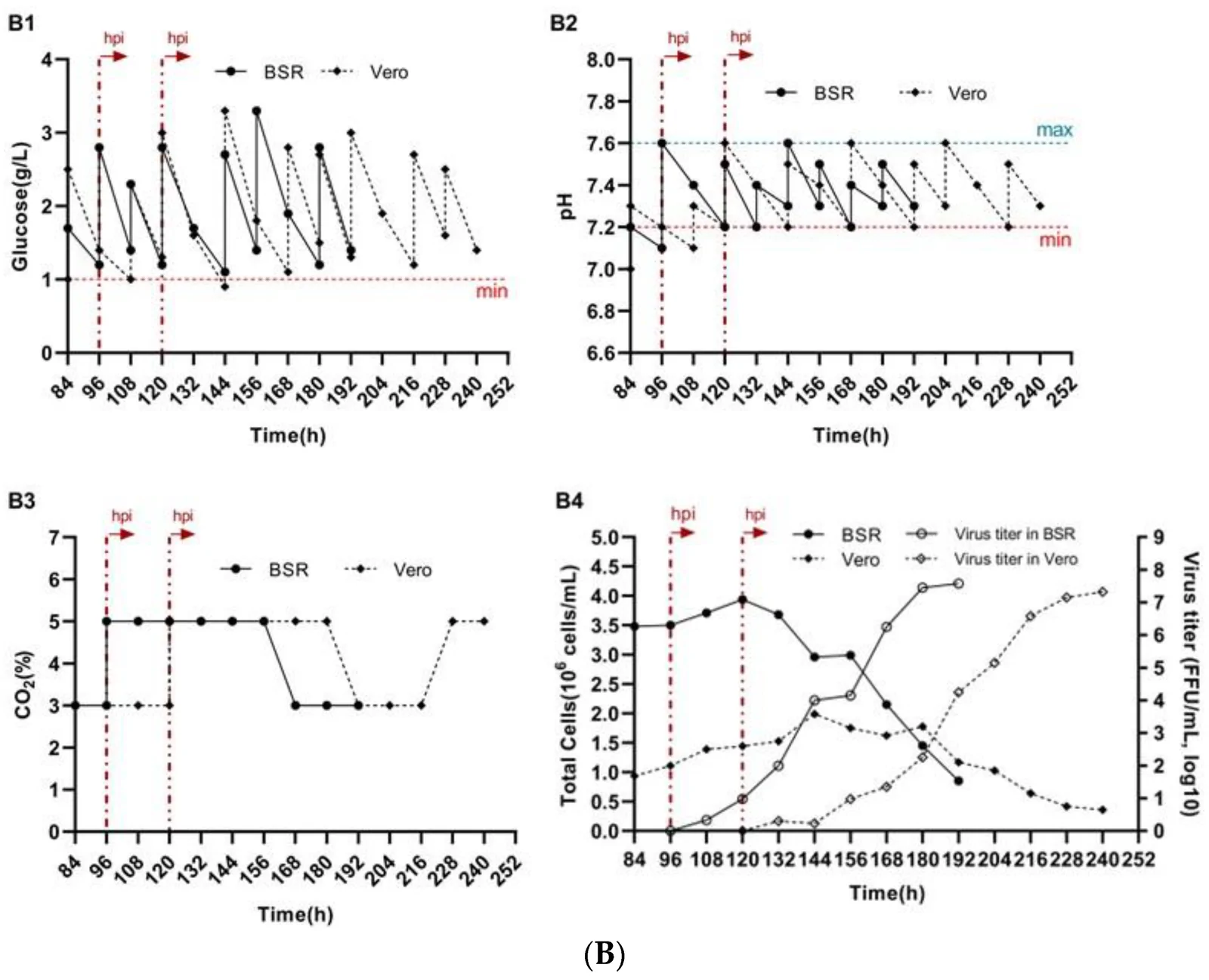
Cultivation of BSR and Vero cells on Cytodex 1 (**A**) and Cytodex 3 (**B**) microcarriers. The dynamics of glucose concentration (**A1**,**B1**), pH of the nutrient medium (**A2**,**B2**), CO_2_ concentration in the incubator atmosphere (**A3**,**B3**), as well as changes in cell density and virus titer (**A4**,**B4**) are shown. In graphs A4 and B4, the infection time (hpi—hours after infection) are indicated by the red dotted line. The upper and lower tolerance limits are indicated by the blue and red dotted lines, respectively. (*) The highest titers of rabies virus were observed on day 4 post-infection in BSR cell cultures and on day 5 in Vero cell cultures.

On day 4 of cultivation, BSR cells that had reached densities of 2.8 × 10^6^ cells/mL with Cytodex 1 and 3.5 × 10^6^ cells/mL with Cytodex 3, as well as Vero cells on day 5 with densities of 1.7 × 10^6^ cells/mL on Cytodex 1 and 2.6 × 10^6^ cells/mL on Cytodex 3, were infected with the rabies virus strain “Rabies virus fix/NIIPBB/2024” at a dose of 0.1 TCID_50_/cell. The virus was pre-diluted at a ratio of 1:100 in fresh culture medium and introduced into the spinner flasks. To maintain stable culture conditions, the glucose concentration and pH of the medium were monitored and adjusted daily. Glucose levels were maintained at 1.0 g/L (Figure 2(A1,B1)), and the pH was kept within the range of 7.2–7.6 (Figure 2(A2,B2)) by regulating the CO_2_ concentration in the incubator (Figure 2(A3,B3)) and periodically supplementing with a 7.0% NaHCO_3_ solution. Following infection, a decrease in the proliferation rate of BSR and Vero cells was observed on both types of microcarriers, while the proportion of infected cells gradually increased. The maximum viral titers in BSR cell cultures were recorded 96 h after virus inoculation and reached 3.3 × 10^7^ FFU/mL for Cytodex 1 and 5.6 × 10^7^ FFU/mL for Cytodex 3 (Figure 2(A4,B4)). In Vero cell cultures, the highest viral titers—2.2 × 10^7^ FFU/mL for Cytodex 1 and 3.8 × 10^7^ FFU/mL for Cytodex 3—were observed 120 h post-inoculation (Figure 2(A4,B4)). Thereafter, a gradual decline in viral productivity was noted, likely attributable to the degradation of the cell population.

### 3.3. Growth of BSR and Vero Cell Lines on BioNOC II™ Macrocarriers

**Figure 3:**
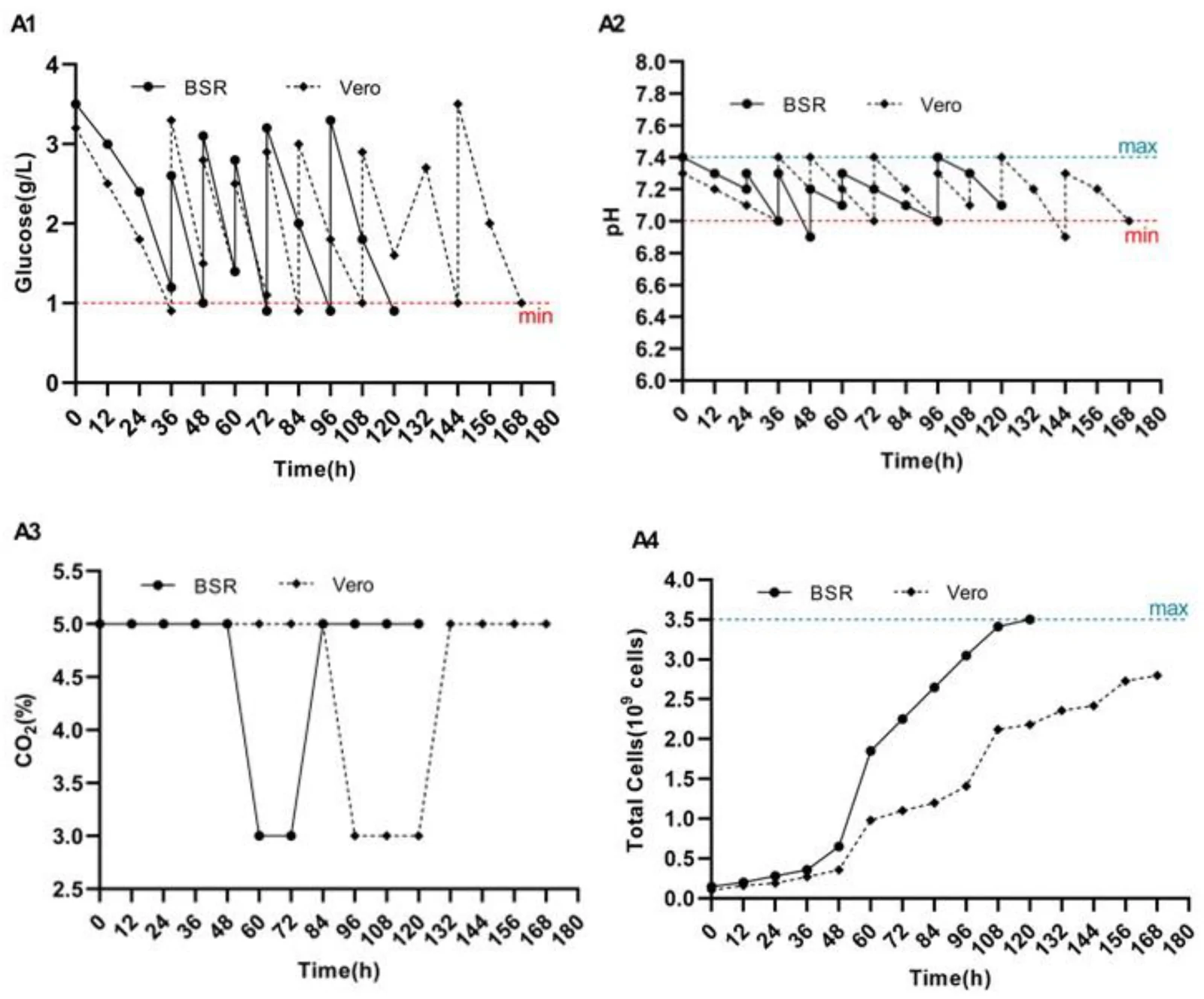
Dynamics of key parameter changes during BSR and Vero cells cultivation on BioNOC II™ macrocarriers. Shown are glucose concentration (**A1**), pH of the culture medium (**A2**), CO_2_ concentration in the incubator atmosphere (**A3**), and the total cell density of BSR and Vero cells (**A4**). Blue and red dotted lines represent the upper and lower permissible limits, respectively.

The cell density in the inocula obtained from culture flasks with a volume of 300 cm^3^ was 2.1 × 10^8^ cells for the BSR culture and 1.5 × 10^8^ cells for the Vero culture. After 5 h of incubation of BelloCell 500A vials in an inverted position at 37 °C in a CO_2_ incubator, the adsorption rates of BSR and Vero cells onto the macrocarriers reached 98% and 95%, respectively. After adsorption, 380 mL of OptiPRO™ SFM medium supplemented with 1% L-glutamine was added to each BelloCell 500A bottle. The white caps were then replaced with blue ones, and the bottles were transferred to the BelloStage™-3000 bioreactor for cultivation. The glucose concentration and pH of the culture medium were carefully monitored and adjusted daily to maintain glucose levels within 1.0–1.5 g/L (Figure 3(A1)) and pH within the range of 7.0–7.4 (Figure 3(A2)). Forty-eight hours after seeding both cells, the growth medium was changed to fresh one. By day 5 of cultivation, the total number of BSR cells reached 3.5 × 10^9^ cells, whereas the maximum density of Vero cells was 2.8 × 10^9^ cells by day 7 of cultivation. The growth kinetics of BSR and Vero cells on BioNOC II™ macrocarriers were assessed at 12-h intervals following the attachment phase (Figure 3(A4)).

### 3.4. Reproduction of Rabies Virus on BioNOC II^®^ Macrocarriers

**Figure 4:**
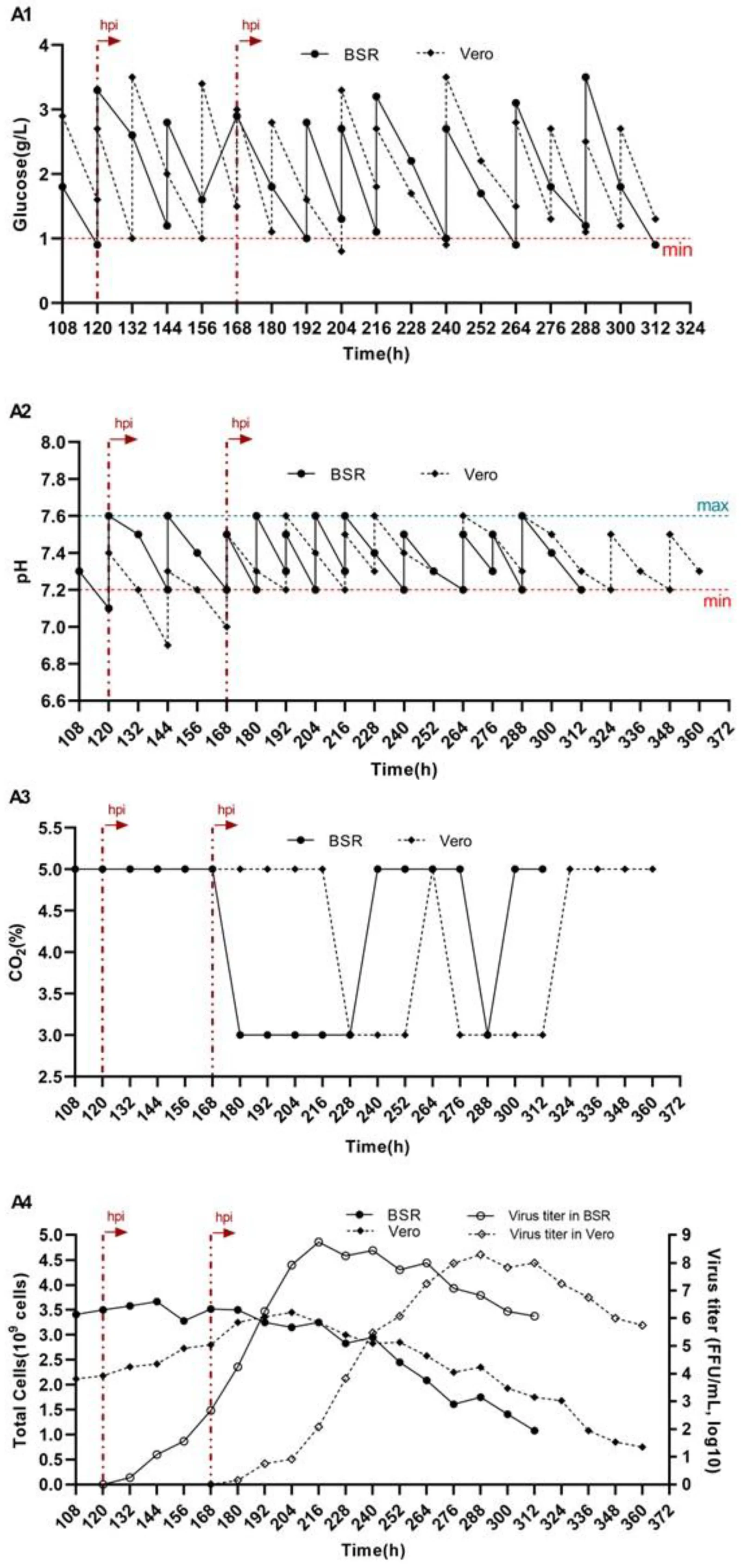
Cultivation of BSR and Vero cells on BioNOC II^®^ macrocarriers. Shown are the dynamics of glucose concentration (**A1**), pH of the culture medium (**A2**), CO_2_ concentration in the incubator atmosphere (**A3**), and changes in BSR and Vero cell density, as well as rabies virus titer (**A4**). The time of infection (hpi—hours post infection) is indicated by a red dotted line. The upper and lower acceptable limits are marked by blue and red dotted lines, respectively. (*) The maximum virus titers were recorded on days 4 and 5 post-infection.

On days 5 and 7 post-seeding, BSR and Vero cells, respectively, were inoculated with the rabies virus strain (Rabies virus fix/NIIPBB/2024) at a dose of 0.1 TCID_50_/cell. During virus inoculation and at 24-h intervals thereafter, the nutrient medium was replaced with fresh medium, and virus-containing supernatants were collected simultaneously. The glucose concentration and pH of the culture medium were monitored daily and adjusted as needed to maintain stable conditions: glucose at a minimum of 1.0 g/L (Figure 4(A1)) and pH within the range of 7.2–7.6 (Figure 4(A2)). Cell density was assessed every 12 h. Cultivation of the rabies virus was terminated upon a marked decline in cell density. Monitoring results indicated a gradual decline in cell concentration beginning 48 h after virus inoculation (Figure 4(A4)). The rabies virus was cultured for 8 days post-infection, with the culture medium completely replaced every 2 days to support optimal conditions for cell viability and viral replication. The maximum virus titers in BSR and Vero cell cultures were recorded on days 4 and 5 post-infection and amounted to 5.6 × 10^8^ FFU/mL and 2.0 × 10^8^ FFU/mL, respectively (Figure 4(A4)). In the subsequent days, a progressive decrease in viral titer was observed.

### 3.5. Impact of Micro—And Macrocarriers on Rabies Virus Titers in BSR and Vero Cells

**Figure 5:**
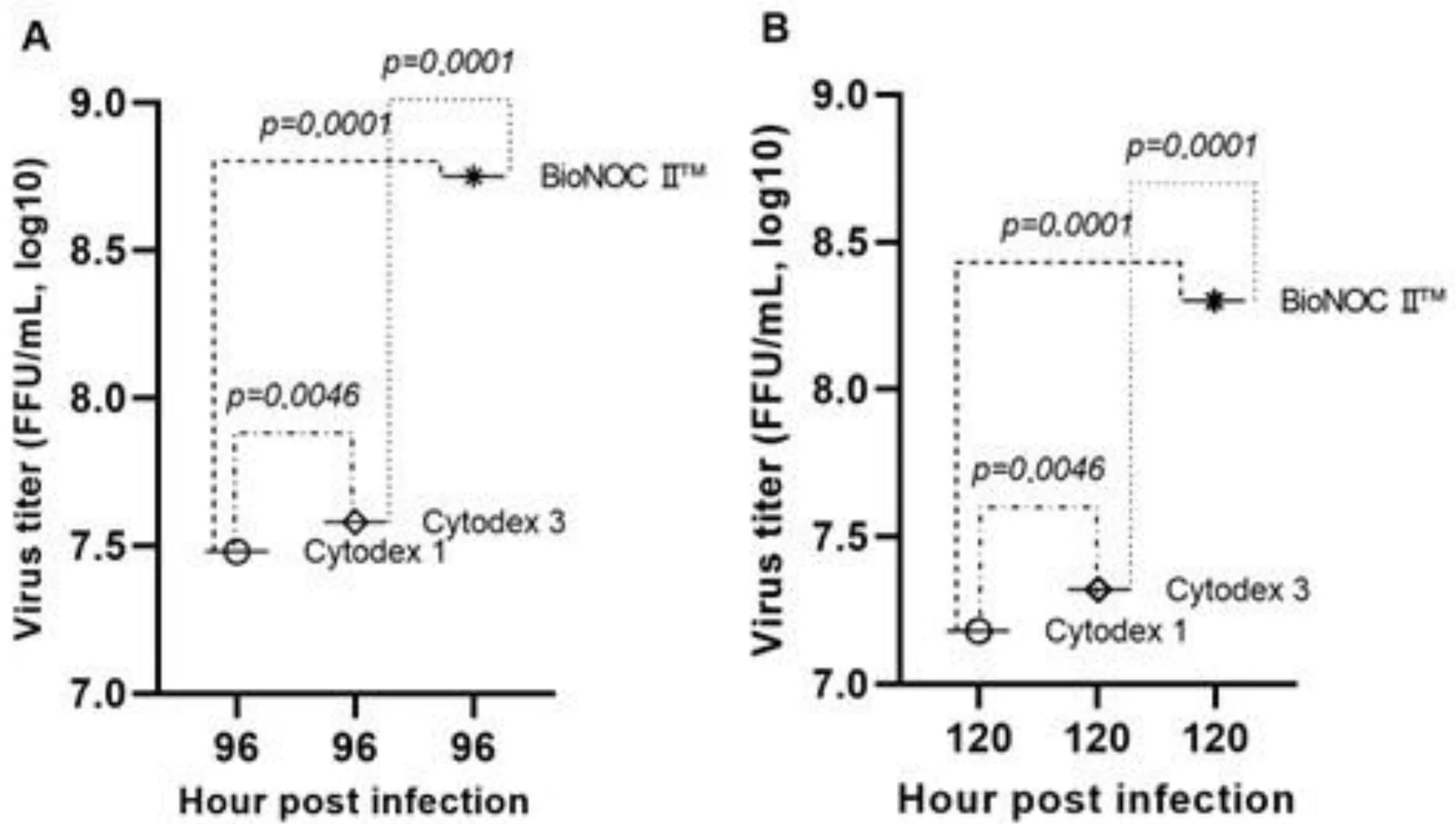
Maximum rabies virus titers in (**A**) BSR and (**B**) Vero cells grown on Cytodex 1, Cytodex 3, and BioNOC II^®^ under serum-free conditions. Statistically significant differences indicate the superior performance of BioNOC II^®^ macrocarriers for viral replication.

Comparison of virus titers obtained on the three types of media revealed statistically significant differences. In BSR cell cultures, the mean virus titer on Cytodex 1 microcarriers was 7.48 ± 0.15 log_10_ FFU/mL, which was significantly lower than that of Cytodex 3 (7.58 ± 0.25 log_10_ FFU/mL; *p* = 0.0046) and BioNOC II^®^ macrocarriers (8.75 ± 0.05 log_10_ FFU/mL; *p* < 0.0001). The difference between the titers obtained with Cytodex 3 and BioNOC II^®^ was also statistically significant (*p* < 0.0001), indicating a significant increase in viral production with the use of macrocarriers (Figure 5A). Similar results were obtained for Vero cells: The mean virus titer on Cytodex 1 microcarriers was 7.18 ± 0.15 log_110_ FFU/mL, which was statistically significantly lower compared to Cytodex 3 (7.32 ± 0.08 log_10_ FFU/mL; *p* = 0.0046) and BioNOC II^®^ (8.30 ± 0.05 log_10_ FFU/mL; *p* < 0.0001). The difference between the titers obtained using Cytodex 3 and BioNOC II^®^ was also statistically significant (*p* < 0.0001), confirming the efficacy of BioNOC II^®^ macrocarriers for rabies virus replication in cell culture (Figure 5B).

The obtained results confirm that the BelloStage™-3000 bioreactor system, when used to culture BSR and Vero cell lines on BioNOC II^®^ macrocarriers in a serum-free medium, enables highly efficient and reproducible rabies virus production, providing a robust foundation for scalable vaccine manufacturing.

## 4. Discussion

The main outcome of this study is the demonstration that the BioNOC II^®^ macrocarrier in combination with the BelloStage™-3000 tide-motion bioreactor provides a highly effective platform for rabies virus production under serum-free conditions. Compared with conventional microcarriers (Cytodex 1 and Cytodex 3), this system supported markedly higher cell proliferation and virus yields, underscoring its superiority for scalable vaccine manufacturing. Importantly, this effect was consistently observed in both BSR and Vero cell cultures, highlighting the robustness of the approach.

The superior performance of the BioNOC II^®^–BelloStage™-3000 system can be explained by a combination of biological and engineering factors. The macroporous structure of BioNOC II^®^ provides a substantially larger surface area for cell attachment than conventional microcarriers, enabling the development of dense, metabolically active biomass. Cells growing within the three-dimensional matrix form stable interactions with the carrier surface, resulting in improved adhesion and culture organization. At the same time, the BelloStage™-3000 system maintains a highly controlled cultivation environment: the gentle tide-motion principle promotes efficient nutrient and oxygen transfer while minimizing shear stress, ensuring uniform distribution of cells and prolonged maintenance of productive infection. These factors collectively account for the markedly enhanced viral titers observed with the macrocarrier–bioreactor system. Furthermore, the use of serum-free media eliminates variability and inhibitory effects associated with serum proteins, improving reproducibility and aligning the process with modern regulatory requirements.

When placed in the context of previous research, the present findings are in line with reports that the type of carrier strongly influences cell proliferation, infection efficiency, and virus yield. For example, several studies have demonstrated that Cytodex 3 typically outperforms Cytodex 1 due to its collagen coating, which facilitates integrin-mediated adhesion and promotes stable cell–cell interactions [31,32]. In our experiments, these trends were reproduced: Cytodex 3 supported higher cell densities and virus titers than Cytodex 1. However, the BioNOC II^®^ macrocarrier–BelloStage™-3000 bioreactor system substantially outperformed both microcarriers, highlighting the advantage of combining advanced carrier design with optimized cultivation technology.

Notably, titers reported in serum-containing systems by other groups [33,34,35] sometimes exceeded those obtained in serum-free settings, reflecting the growth-promoting effects of serum-derived factors. For instance, high virus yields have been achieved with BHK-21 and Vero cells cultured on Cytodex 3 microcarriers in media supplemented with fetal bovine serum. However, such approaches face significant drawbacks, including batch-to-batch variability, high cost, limited reproducibility, and ethical concerns. In this context, the ability of the BioNOC II^®^–BelloStage™-3000 system to achieve yields comparable to, or even higher than, serum-dependent systems represent a substantial step forward. Comparable success of the BelloStage™ platform has also been reported for other viruses, such as SARS-CoV-2, influenza A, Japanese encephalitis virus, Rift Valley fever virus, and others [30,36,37,38,39,40]. Together, these findings confirm the broad applicability and versatility of this technology.

From an industrial perspective, the BelloStage™-3000 bioreactor is a laboratory-scale model of the scalable TideXcell™ platform, which allows direct linear scale-up from volumes of 0.5 L to 100 L without the need for major process modifications. This scalability, combined with serum-free operation, provides a pathway for the seamless transfer of optimized laboratory protocols into industrial production. The design of the BioNOC II^®^–BelloStage™-3000 system not only facilitates high reproducibility but also simplifies downstream processing by reducing serum-derived impurities, thereby easing regulatory approval pathways. These features make the platform particularly attractive for vaccine manufacturing in low- and middle-income countries, where cost-effectiveness, safety, and robustness are critical considerations [41].

Despite the promising results, several limitations should be noted. Experiments were performed only at laboratory scale, using two cell lines (BSR and Vero), and the immunogenicity and protective efficacy of the produced virus were not evaluated in vivo. Additionally, potential differences in genome sequence, antigenicity, or expression of the major antigenic protein (G protein) among viruses propagated on different carriers were not assessed. Future studies should address these aspects, including comparative analyses of G protein expression and antigenicity, to fully evaluate the suitability of the BioNOC II^®^–BelloStage™-3000 system for scalable rabies vaccine production with preserved immunogenic properties.

Future studies should therefore focus on (i) validation of scale-up to pilot and industrial volumes using the TideXcell™ system, (ii) comparative benchmarking against other emerging bioreactor platforms and carrier types to optimize yield and cost-effectiveness, and (iii) evaluation of long-term stability, safety, and immunogenicity of rabies virus produced under serum-free conditions. Addressing these aspects will be crucial for translating the BioNOC II^®^–BelloStage™-3000 platform from laboratory research into routine vaccine manufacturing and for supporting the development of modern, GMP-compliant, and globally accessible rabies vaccines.

Future Perspectives

The BelloStage™-3000 system can be further explored for large-scale commercial vaccine production, demonstrating suitability for industrial applications, particularly in low- and middle-income countries where cost-effectiveness, reproducibility, and biosafety are essential. Comparative studies with other emerging bioreactor platforms and advanced carrier types could help optimize viral yield, scalability, and overall cost-effectiveness. Benchmarking these systems will identify the most efficient combinations of bioreactor design and carrier properties for rabies virus production.

Studies focusing on the long-term stability, safety, and immunogenicity of rabies virus produced using the BioNOC II^®^–BelloStage™-3000 platform are critical before clinical translation. Addressing these aspects is crucial for translating the platform from laboratory research into routine vaccine manufacturing and supporting the development of modern, GMP-compliant, and globally accessible rabies vaccines.

## 5. Conclusions

The results demonstrate that rabies virus replication efficiency is strongly influenced by the choice of cultivation system and carrier. BioNOC II^®^ macrocarriers in the laboratory-scale BelloStage™-3000 bioreactor yielded the highest virus titers, with BSR cells outperforming Vero cells in productivity, supporting their use as a promising alternative substrate for industrial rabies vaccine production. The Tide Motion operation ensures efficient nutrient and oxygen transfer and maintains stable microenvironmental parameters, which, together with BioNOC II^®^ macrocarriers, significantly enhance process efficiency compared to Cytodex 1 and Cytodex 3 microcarriers. This approach establishes a state-of-the-art technology platform combining high efficiency, reproducibility, and scalability, suitable for industrial process transfer and the development of veterinary rabies vaccines.

## Data Availability

The original contributions presented in the study are included in the article/Supplementary Materials, further inquiries can be directed to the corresponding authors.

## Supplementary Materials

The following supporting information can be downloaded at: https://www.mdpi.com/article/10.3390/biology14101455/s1.
