# Correction: Amanova et al. Efficient Serum-Free Rabies Virus Propagation Using BSR and Vero Cell Lines: A Comparative Evaluation of BioNOC II^®^ Macrocarriers in the BelloStage™-3000 Bioreactor Versus Conventional Microcarriers. *Biology* 2025, *14*, 1455

**DOI:** 10.3390/biology15141153

**Published:** 2026-07-15

**Authors:** Zhanat Amanova, Zhanna Sametova, Sholpan Turyskeldy, Alina Kurmasheva, Ruslan Abitayev, Abdurakhman Ussembay, Zhanat Kondibayeva, Dariya Toktyrova, Dana Mazbayeva, Sergazy Nurabayev, Aslan Kerimbayev, Yerbol Bulatov

**Affiliations:** 1 Research Institute for Biological Safety Problems, National Holding “QazBioPharm”, Gvardeiskiy 080409, Kazakhstan; zh.sametova@biosafety.kz (Z.S.); sh.smankizi@biosafety.kz (S.T.); a.kurmasheva@biosafety.kz (A.K.); r.abitaev@biosafety.kz (R.A.); a.ussenbay@biosafety.kz (A.U.); zh.kondybaeva@biosafety.kz (Z.K.); d.toktirova@biosafety.kz (D.T.); d.mazbayeva@biosafety.kz (D.M.); s.nurabayev@biosafety.kz (S.N.); a.kerimbayev@biosafety.kz (A.K.); 2 Faculty of Biology and Biotechnology, Department of Biotechnology, Al-Farabi Kazakh National University, Almaty 050040, Kazakhstan

**Additional Affiliation**

In the published publication [1], there was an error regarding the affiliation for Dariya Toktyrova. In addition to affiliation 1, the updated affiliations should include the following: Faculty of Biology and Biotechnology, Department of Biotechnology, Al-Farabi Kazakh National University, Almaty 050040, Kazakhstan. The authors state that the scientific conclusions are unaffected. This correction was approved by the Academic Editor. The original publication has also been updated.
