# Supplementary material for: Efficient Serum-Free Rabies Virus Propagation Using BSR and Vero Cell Lines: A Comparative Evaluation of BioNOC II® Macrocarriers in the BelloStage™-3000 Bioreactor Versus Conventional Microcarriers
Source: Biology (Basel). 2025 Oct 21;14(10):1455. doi: 10.3390/biology14101455 (PMC12561483; doi:10.3390/biology14101455)
Supplement: Supplementary file 1 [file biology-14-01455-s001.zip › Supplementary Materials Techne Magnetic Stirrer.pdf]

Supplementary Materials

Title: *Detailed Protocol for Cultivation of BSR and Vero Cells on Microcarriers Using a Techne Magnetic Stirrer*

Description:

This supplementary document provides the complete methodology for culturing BSR and Vero cell lines on Cytodex 1 and Cytodex 3 microcarriers using a 250 mL Techne spinner (Bibby Scientific, UK) at 37 °C in a humidified incubator with 5% CO_2.

The protocol includes:

• Preparation of inoculum and seeding of spinners at a density of ≥3,0 × 10^5 cells/mL.

• Addition of Cytodex 1 or Cytodex 3 microcarriers at 2,0 g/L.

• Initial cell attachment period with intermittent stirring (2 min stirring / 10 min pause) followed by continuous stirring at 60 rpm.

• Daily monitoring of culture conditions, including pH (maintained at 7,0–7,4) and glucose concentration (maintained ≥1,0 g/L).

• Medium replacement schedule: 60% replacement with fresh medium after 48 h to restore nutrients.

• Daily assessment of cell growth dynamics via sampling and determination of cell concentration.

• Cell detachment from microcarriers using 2 M NaCl solution.

• Viability assessment using trypan blue exclusion (0,4%) and counting with a TC20 automated cell counter (Bio-Rad, USA).

Notes:

• Inoculum doses, stirring speeds, and glucose adjustments were based on preliminary optimization to ensure efficient virus replication, maintain cell viability, and support metabolic requirements.

• This document provides operational details that complement the abbreviated methodology described in the main manuscript.

Link to main manuscript: Section 2.3 "*Cultivation of BSR and Vero Cells on a Techne Magnetic Stirrer*"
