# Supplementary material for: Efficient Serum-Free Rabies Virus Propagation Using BSR and Vero Cell Lines: A Comparative Evaluation of BioNOC II® Macrocarriers in the BelloStage™-3000 Bioreactor Versus Conventional Microcarriers
Source: Biology (Basel). 2025 Oct 21;14(10):1455. doi: 10.3390/biology14101455 (PMC12561483; doi:10.3390/biology14101455)
Supplement: Supplementary file 1 [file biology-14-01455-s001.zip › Supplementary Materials Rabies Virus Inoculation.pdf]

Supplementary Materials

Title: *Detailed Protocol for Inoculation and Proliferation of the Rabies Virus on Microcarriers Using a Techne Magnetic Stirrer*

Description:
This supplementary document provides the complete methodology for inoculating and propagating the rabies virus in BSR and Vero cell lines cultured on Cytodex 1 and Cytodex 3 microcarriers using a Techne magnetic stirrer.

The protocol includes:

- Pausing the magnetic stirrer for 10 min to allow microcarriers to sediment once cells reached a density of 1,5–2,5 × 10^6 cells/mL.
- Replacing two-thirds of the spent OptiPRO™ SFM medium with fresh medium containing the rabies virus at a dose of 0,1 $TCID_{50}$/mL.
- Facilitating virus adsorption by incubating under intermittent stirring (2 min agitation / 10 min pause) for 30 min.
- Resuming continuous stirring at 50–60 rpm for subsequent cultivation.
- Incubation in a CO_2 incubator (Nüve, Turkey) at 37 °C with 5% CO_2.
- Replacing the culture medium after 48 h with fresh medium to maintain nutrient levels.
- Daily monitoring of cell concentration, pH (7,2–7,6), and glucose content (≥1,0 g/L).
- Harvesting viral suspension using the freeze–thaw method once 80–90% of cells were infected.

Notes:

- Detailed operational parameters, including stirring speeds and adsorption conditions, are provided in this document.
- This supplementary material complements the abbreviated methodology described in Section 2.4 of the main manuscript.

Link to main manuscript: Section 2.3 "*Cultivation of BSR and Vero Cells on a Techne Magnetic Stirrer*"
