# Supplementary material for: Efficient Serum-Free Rabies Virus Propagation Using BSR and Vero Cell Lines: A Comparative Evaluation of BioNOC II® Macrocarriers in the BelloStage™-3000 Bioreactor Versus Conventional Microcarriers
Source: Biology (Basel). 2025 Oct 21;14(10):1455. doi: 10.3390/biology14101455 (PMC12561483; doi:10.3390/biology14101455)
Supplement: Supplementary file 1 [file biology-14-01455-s001.zip › Supplementary Materials Rabies BelloStage.pdf]

Supplementary Materials

Title: *Detailed Protocol for Inoculation and Propagation of Rabies Virus on BioNOC II® Macrocarriers Using the BelloStage™-3000 System*

Description:

This supplementary document provides the complete methodology for inoculating and propagating rabies virus on BSR and Vero cells cultured on BioNOC II® macrocarriers using the BelloStage™-3000 system.

The protocol includes:

Cell Preparation:

- Seed BSR and Vero cells on BioNOC II® macrocarriers at a density of 1,5–3,0 × 10^8 cells per flask.

2. Virus Inoculation:

- Add 500 mL of rabies virus inoculum (Rabies virus fix/NIIPBB/2024) at a dose of 0,1 $TCID_{50}$/mL to the culture bottle.

3. Initial Virus Adsorption:

- Incubate the BelloCell 500A culture bottle for 1 h at 37 °C in the BelloStage™-3000 system under the following conditions:
  - Lift speed: 2.0 mm/s
  - Top hold time (T_H): 20 s
  - Lowering speed: 2.0 mm/s
  - Bottom hold time (B_H): 0 s

4. Cultivation Post-Adsorption:

- After 1 h, switch the BelloStage™-3000 system to the following parameters for continued cultivation:
  - Lift speed: 1.0 mm/s
  - Top hold time (T_H): 10 s
  - Lowering speed: 1.0 mm/s
  - Bottom hold time (B_H): 10 s

5. Monitoring:

- Collect samples daily to monitor pH (7,2–7,6) and glucose concentration (≥1,0 g/L) in the medium.

Notes:

- Detailed operational parameters, including lift speeds, hold times, and sampling schedules, are provided in this document.
- This supplementary material complements the abbreviated methodology described in Section 2.6 of the main manuscript.

Link to main manuscript: Section 2.6 "Inoculation and Propagation of Rabies Virus on BioNOC II® Macrocarriers"
