# Supplementary material for: Efficient Serum-Free Rabies Virus Propagation Using BSR and Vero Cell Lines: A Comparative Evaluation of BioNOC II® Macrocarriers in the BelloStage™-3000 Bioreactor Versus Conventional Microcarriers
Source: Biology (Basel). 2025 Oct 21;14(10):1455. doi: 10.3390/biology14101455 (PMC12561483; doi:10.3390/biology14101455)
Supplement: Supplementary file 1 [file biology-14-01455-s001.zip › Supplementary Materials BelloStage.pdf]

Supplementary Materials

Title: *Detailed Protocol for Cultivation of BSR and Vero Cells in the BelloStage™-3000 Bioreactor System*

Description:

This supplementary document provides the complete methodology for culturing BSR and Vero cells on BioNOC II® macrocarriers in the BelloStage™-3000 bioreactor system using OptiPRO™ SFM serum-free medium supplemented with L-glutamine, penicillin, and streptomycin.

The protocol includes:

1. Initial cell culture:

BSR and Vero cells were initially cultured in 300 cm^3 culture flasks (#90300, Techno Plastic Products AG, Switzerland) using OptiPRO™ SFM supplemented with L-glutamine.

2. Cell dissociation:

- Wash the cell monolayer twice with PBS (#10010023, Gibco™, USA).
- Add 0,25% trypsin-EDTA (1X) (#25200056, Gibco™, USA) and incubate for 3 min at 37 °C in a CO_2 incubator.

3. Cell preparation:

- Centrifuge detached cells at 180 g for 5 min and resuspend in 20 mL of fresh OptiPRO™ SFM supplemented with L-glutamine, penicillin, and streptomycin.
- Resuspend cells in 20 mL fresh OptiPRO™ SFM supplemented with L-glutamine, penicillin, and streptomycin.
- Adjust cell concentration to $\geq 1{,}5 \times 10^7$ cells/mL.

4. Inoculation into macrocarriers:

- Add 20 mL of cell suspension to BelloCell 500A flasks containing BioNOC II® macrocarriers pre-treated with 100 mL of medium.
- Seal flasks with white caps and place upside down in a 37 °C incubator.
- Gently mix periodically: every 15 min during the first hour and every 30 min during the next 4 h to allow cells to attach to macrocarriers.
- Ensure macrocarriers are fully submerged in the cell suspension.

5. Preparation for BelloStage™-3000 System:

- After cell attachment, add 380 mL of fresh OptiPRO™ SFM with L-glutamine to the flasks.
- Replace the white cap with a blue cap containing a 0,22 μm filtration membrane.

6. Cultivation in BelloStage™-3000:

- Transfer the flasks to the BelloStage™-3000 system.
- Culture cells under the following original system parameters (not modified):
  - Lift speed: 1,5 mm/s
  - Top hold time (T_H): 0 s
  - Lower speed: 1,5 mm/s
  - Bottom hold time (B_H): 90 s
- Replace 60% of the medium with fresh medium after 48 h.
- Monitor cell density, pH (7,0–7,4), and glucose concentration (≥1,0 g/L) daily.

Notes:

- Detailed operational parameters, including lift speeds, hold times, and mixing schedules, are provided in this document.
- This supplementary material complements the abbreviated methodology described in Section 2.5 of the main manuscript.

Link to main manuscript: Section 2.5 "*Cultivation of BSR and Vero Cells in the BelloStage™-3000 Bioreactor System*"
